# Does Animal-Mediated Seed Dispersal Facilitate the Formation of *Pinus armandii*-*Quercus aliena* var. *acuteserrata* Forests?

**DOI:** 10.1371/journal.pone.0089886

**Published:** 2014-02-28

**Authors:** Fei Yu, Dexiang Wang, Xianfeng Yi, Xiaoxiao Shi, Yakun Huang, Hongwu Zhang, XinPing Zhang

**Affiliations:** 1 College of Forestry, Northwest A & F University, Yangling, Shaanxi, China; 2 College of Life Sciences, Jiangxi Normal University, Nanchang, Jiangxi, China; 3 College of Animal Science and Technology, Northwest A & F University, Yangling, Shaanxi, China; DOE Pacific Northwest National Laboratory, United States of America

## Abstract

The *Pinus armandii* and *Quercus aliena* var. *acuteserrata* mixed forest is one of the major forest types in the Qinling Mountains, China. *P. armandii* is considered to be a pioneer species during succession and it is usually invaded by late successional *Q. aliena* var. *acuteserrata*. However, the mechanism that underlies its invasion remains unclear. In the present study, we tracked seed dispersal of *P. armandii* and *Q. aliena* var. *acuteserrata* using coded plastic tags in the western, middle and eastern Qinling Mountains to elucidate the invasion process in the mixed forests. Our results indicated that the seeds of both *P. armandii* and *Q. aliena* var. *acuteserrata* were removed rapidly in the Qinling Mountains, and there were no differences in the seed removal rates between the two species. There were significant differences in rodent seed-eating and caching strategies between the two tree species. For *P. armandii*, seeds were more likely to be eaten in situ than those of *Q. aliena* var. *acuteserrata* in all plots. By contrast, the acorns of *Q. aliena* var. *acuteserrata* were less frequently eaten in situ, but more likely to be removed and cached. *Q. aliena* var. *acuteserrata* acorns had significantly longer dispersal distances than *P. armandii* seeds in all plots. Although *P. armandii* seeds were less likely to be dispersed into the *Q. aliena* var. *acuteserrata* stands, over 30% of the released acorns were transported into the *P. armandii* stands where they established five seedlings. Based on the coupled recruitment patterns of *P. armandii* and *Q. aliena* var. *acuteserrata*, we suggest that the animal-mediated seed dispersal contributes to the formation of *Pinus armandii*-*Quercus aliena* var. *acuteserrata* forests.

## Introduction

Natural forest regeneration and succession may depend on the arrival of animal- or wind-dispersed seeds [1]–[4]. Many rodent species, as well as jays, play important roles in the secondary dispersal of pine or oak species via their hoarding behaviors in temperate zonal forests [5]–[7]. Seed removal by rodents has indirect effects on regeneration and colonization in plant populations, thereby affecting plant community structure [8]. It is generally believed that *Quercus* can colonize the understory of pine forests via the jay- or rodent-mediated dispersal of acorns [9]–[11]. Oaks depend entirely on animals for the dispersal of their seeds because they have not evolved wind-dispersal structures [12]. By contrast, most *Pinus* species rely on seed dispersal by wind, which is facilitated by their small seeds with attached wings [13]–[15]. However, some *Pinus* species (e.g., *Pinus armandii* and *Pinus koraiensis*) depend on animals for seed dispersal [16]–[18]. It is not clear whether these animal-dispersed *Pinus* species can colonize or establish successfully in the understory of oak forests.

It is widely accepted that the successional changes in pine-oak forests follow the patterns of vegetation dynamics described for the Mediterranean Basin, where *Pinus* species are considered to be pioneer species during succession, and are usually invaded by late-successional *Quercus* species [9], [19]. However, the mechanism that underlies the invasion is not well understood. In this framework, mixed forests are usually considered to be a successional stage of initial post-disturbance pine forests [19]–[21]. Evidence has accumulated that pine recruits occur at a higher frequency and at a greater abundance under the canopies of their congeners, whereas oak species usually exhibit greater regeneration than pine species in pine-dominated stands and mixed oak–pine forests [22]–[24]. In central China, the *Pinus armandii* and *Quercus aliena* var. *acuteserrata* mixed forest is one of the major forest types in the Qinling Mountains [21], [25]. Studies have shown that oak seedlings had a comparable abundance to pine seedlings in *P. armandii* forests [21], [26]. By contrast, *P. armandii* seedlings are rare in oak forests in the mosaic distribution regions between *P. armandii* and *Q. aliena* var. *acuteserrata* forests [21], [27]. The explanation for this phenomenon remains unclear and we have little knowledge of how the seed dispersal of these two species contributes to this phenomenon. Moreover, few studies have focused on how *P. armandii* is invaded by the late successional *Q. aliena* var. *acuteserrata* in the oak-pine forest belt.

In the present study, we tracked individual seeds of *P. armandii* and *Q. aliena* var. *acuteserrata* using coded plastic tags and explored the effects of small rodents on the dispersal of the seeds of these two species in the middle, eastern and western Qinling Mountains. The aims of this study were as follows: (1) To explain the patterns where oak seedlings are often observed in the *P. armandii* forest, whereas *P. armandii* seedlings are seldom found in oak forests in the mosaic distribution regions. (2) To explore why *P. armandii* stands are usually invaded by late successional *Quercus aliena* var. *acuteserrata*. We hypothesized that the rodent-mediated seed dispersal of *Q. aliena* var. *acuteserrata* into *P. armandii* stands might explain the succession mechanism in *Pinus armandii*-*Quercus aliena* var. *acuteserrata* mixed forests in the Qinling Mountains, China. Thus, we aimed to elucidate the succession mechanism in oak-pine mixed forests and provide valuable information to support the development of effective forest management and restoration plans.

## Materials and Methods

### Ethics statement

This study was carried out in strict accordance with the current laws of China. The protocol was approved by the Administrative Panel on the Ethics of Huoditang Forest, Northwest A & F University. We signed a contract with the Huoditang Forest in 2012, and the contract included the permissions to access the study site and conduct this study.

### Study site

We conducted the experiments on south-facing slopes within Qinling Mountains. Three experimental plots were established in (1) the western region of the Qinling Mountains (WQ) on Xiaolong Mountain, Gansu Province, (2) the middle region of the Qinling Mountains (MQ) at the Qinling National Forest Ecosystem Research Station in Huoditang Forest, Ningshaan County, Shaanxi Province, and (3) the eastern region of the Qinling Mountains (EQ) in Luonan County, Shaanxi Province (Table 1). Three identical experiments were carried out in these three plots to test the same question in different regions of the mountains. The Qinling Mountains run east–west and form the basin divider between the two longest rivers in China, the Yellow River and the Yangtze River. The Qinling Mountains are situated in the transitional zone between two macroclimatic regimes (subtropical and warm-temperate zones) where the annual precipitation ranges from 950 to 1,200 mm, most of which falls between July and September. Snow cover usually lasts five or more months (from November to March), and the mean annual temperature ranges from 6 to 11°C below 2,000 m and from 1 to 6°C above 2,000 m above sea level [28]. The natural vegetation types in the Qinling Mountains are deciduous broad-leaved forests (below 2,000 m), mixed conifer and deciduous forests (800–2,500 m), and conifer forests (above 2,500 m). The dominant tree species are *Q. aliena* var. *acuteserrata*, *P. armandii*, *Betula albosinensis*, *B. luminifera*, *P. tabulaeformis*, *Picea wilsonii*, *Abies fargesii*, *Populus davidiana*, *Toxicodendron vernicifluum*, and *Acer davidii*. The *P. armandii* and *Q. aliena* var. *acuteserrata* mixed forest belt covers about a quarter of the Qinling Mountains. Several rodent species coexisted in the study site and the dominant species are *Apodemus draco*, *Sciurotamias davidianus* and *Niviventer confucianus*. Previous studies show that these rodents are likely to affect the natural regeneration of the main tree species [17], [29].

**Table 1:** Characteristics of the three experimental plots.

Study site	Longitude (E)	Latitude (N)	Location in China	Altitude (m.a.s.l.)	Average annual temperature (°C)	Average annual rainfall (mm)	Dominant species
MQ	108°21′–108°39′	33°18′–33°28′	Ningshaan County, Shaanxi	1,470–2,473	12.7	1,130	*Quercus aliena* var. *acuteserrata*, *Pinus armandii*, *Pinus tabulaeformis*, *Betula albosinensis*
EQ	109°44′–110°40′	33°52′–34°25′	Luonan County, Shaanxi	800–1,200	11.5	779	*Quercus aliena* var. *acuteserrata*, *Pinus armandii*, *Pinus tabulaeformis*, *Quercus variabilis*
WQ	104°22′–106°43′	33°30′–34°49′	Tianshui City, Gansu	700–3,330	10.9	518.5	*Quercus aliena* var. *acuteserrata*, *Pinus armandii*, *Pinus tabulaeformis*, *Quercus liaotungensis*

WQ: study area located in the western Qinling Mountains; MQ: study area located in the middle Qinling Mountains; EQ: study area located in the eastern Qinling Mountains.

### Identification of seed removers

To determine the abundance of rodents that could potentially remove the released seeds, 50 live steel-wire traps (30 cm×25 cm×20 cm) baited with peanuts were placed in each plot along each of the two transects at an interval of 5 m apart during October 8–11, 2012 (MQ, EQ and WQ). The traps were checked twice a day at sunrise and sunset. The captured animals were weighed and released immediately in situ. We did not mark the captured animals to identify recaptures. Trapping was conducted for three consecutive days. The total number of trapping days and nights was 150 (50 traps×3 days and nights) for each plot.

### Seed dispersal experiment

We selected plots that each measured about 3.0 ha at the experimental plots in MQ, EQ, and WQ, i.e., the western, eastern and middle regions of the Qinling Mountains, respectively. Mature and fresh seeds of *Q. aliena* var. *acuteserrata* and *P. armandii* were collected from the ground outside our experimental plots for field release during the first fortnight of October 2012. We used water flotation to distinguish between sound and insect-damaged/empty seeds. We randomly selected 600 fresh sound seeds of *P. armandii* seeds (1.30×0.78 cm, 0.36±0.03 g, n = 100) and *Q. aliena* var. *acuteserrata* acorns (1.69×1.40 cm, 1.70±0.04 g, n = 100) from each of the three plots, respectively (in total 3,600 seeds), and labeled them using slight modifications of the methods reported by Zhang and Wang [30] and Li and Zhang [31]. A tiny hole measuring 0.3 mm in diameter was drilled through the husk near the germinal disc of each seed, without damaging the cotyledon and the embryo. A white flexible plastic tag (3.0×1.0 cm, <0.1 g) was tied through the hole in each seed using a thin 10 cm long steel thread. To ensure that each seed could be relocated and identified easily, each seed was numbered consecutively and discriminatively with a tag. When rodents buried the seeds in the soil or litter, the tags were often still visible on the surface of the ground, which made them easy to find. Tagging has been shown to have a negligible effect on seed removal and hoarding by rodents [30], [32].

**Figure 1:**
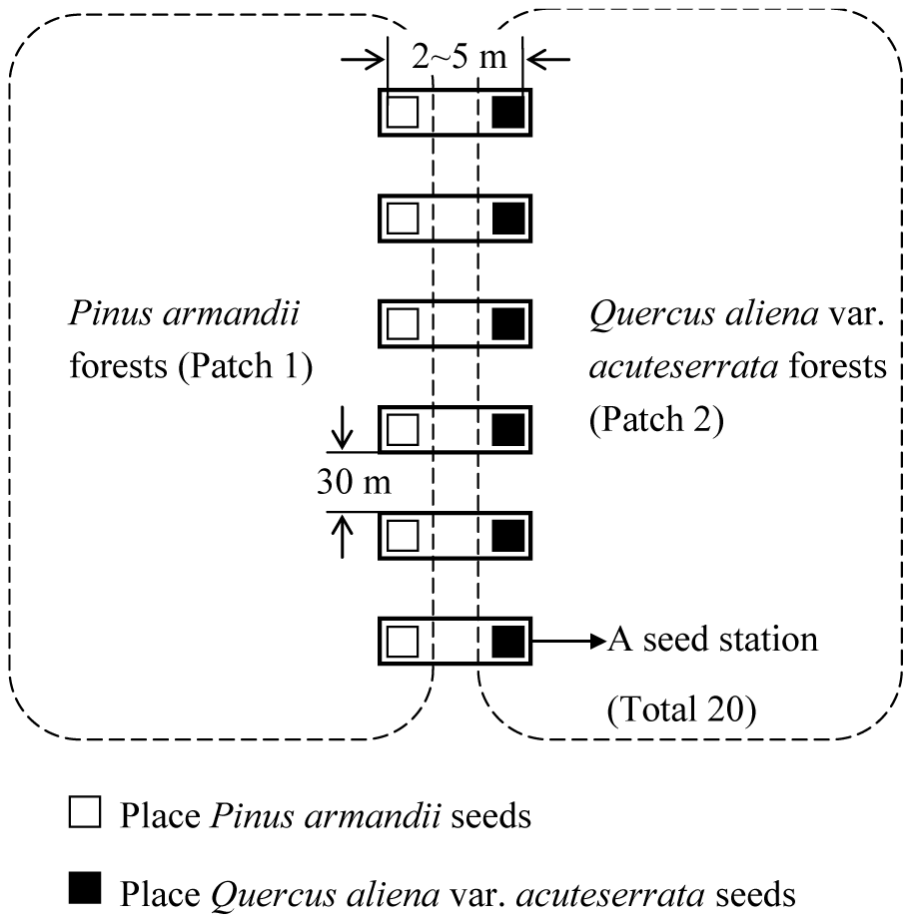
Sketch map of the locations of seed stations in the experimental plots.

In each plot, 20 seed stations were established on the forest edges between *P. armandii* and *Q. aliena* var. *acuteserrata* forests, spaced 30 m apart along a transect line (Fig. 1). We used Whitmore's [33] characterization of the forest mosaic to distinguish between two types of patches, i.e., *P. armandii* forests dominated by *P. armandii* (patch 1) and *Q. aliena* var. *acuteserrata* forest dominated by *Q. aliena* var. *acuteserrata* (patch 2). We placed 30 tagged seeds of each species in separate batches (2–5 m apart) at each seed station (Fig. 1). The total number of seeds released was 20 (stations)×30 (seeds)×2 (species)×3 (plots) = 3,600 seeds. Starting the day after placement, we checked for seed removal on a daily basis until all of the seeds were removed or consumed. During each visit, we randomly searched the area around each seed station and recorded the status of all released seeds. The post-dispersal seed fates were classified using six categories: 1) intact in situ (IS); 2) eaten in situ (EIS); 3) eaten after removal (EAR); 4) intact after removal to another location (IAR); 5) cached after removal (CAR); and 6) missing where their true fates were unknown (M). When a cache was discovered, we carefully recorded the seed code numbers, measured the distance of the tagged seeds from their original seed stations, and determined the cache location using a chopstick to mark the cache location, which was coded with the same number as the tag. The sticks were placed 25 cm from the seed caches. If the seeds were scatter-hoarded, we recorded whether they were cached under the canopy of *P. armandii* or *Q. aliena* var. *acuteserrata*. During the next visit, we also checked all of the caches that were relocated in previous visits until the caches were removed or eaten by rodents. If a marked cache was removed, the area around the cache (radius <30 m) was searched randomly. These experiments were conducted during October 15–November 17, 2012. Seed germination was surveyed during the following spring in 2013.

### Seed rain survey

We measured the seed rain of *Q. aliena* var. *acuteserrata* and *P. armandii* forests using three plots (separate plots in MQ, EQ, and WQ). During 2012, three 500 m baseline transects were established in the mosaic distribution regions between *Q. aliena* var. *acuteserrata* and *P. armandii* forests in MQ, EQ, and WQ, respectively. In each plot, seed traps were placed along eight parallel transects (10 traps spaced 20 m apart along each transect), which were separated by 20 m perpendicular to the baseline, on both sides of the forest edge (n = 4). Transects on the west and east sides of the forest edge were *P. armandii* forest (patch 1) and *Q. aliena* var. *acuteserrata* forest (patch 2), respectively. Thus, the total number of traps = 40 traps×2 patches = 80 in each plot.

The seed traps were hung from the lower branches of trees to avoid predation by terrestrial vertebrate predators. The traps were located randomly and specially designed to capture seeds. Avian predation was regarded as having a less significant effect on potential seed losses because few bird species was observed in the experimental plots during our surveys. A 1×1.2 m polyester net (1 mm mesh) was fastened to a 0.5 m^2^ metal frame, which formed a concave seed trap with an aperture of 0.5 m^2^ and a depth of 0.3 m, thereby preventing the seeds from rebounding after falling. The frame was set on a thin wooden rod about 1.2 m above the ground to prevent predation by terrestrial vertebrates. The traps were used to catch seeds and other debris while letting rainfall pass through easily. The traps were left in place until all of the ripe seeds had fallen from the trees. All of the seeds captured from trees were counted and identified in the laboratory.

### Data analysis

SPSS for Windows (Version 17.0) was used for the statistical analyses. One-way ANOVA was used to detect the difference in the seed crops among three plots. The proportions of remaining, eaten, and cached seeds were arcsine square root transformed before the statistical analysis. Cox regression was used to test for differences in the seed removal rates between the two species. Two-way ANOVA was used to test for difference in the seed dispersal distances and the proportion of the seed fates between *P. armandii* and *Q. aliena* var. *acuteserrata*.

## Results

### Rodent abundance and seed availability

Although several traps triggered false, a total of 23, 44, and 37 rodents were captured in WQ, MQ, and EQ, respectively (Table 2). In WQ, four rodent species were trapped during 150 trap nights. By contrast, only three species were captured in MQ and EQ (Table 2).

**Table 2:** Number of small rodents captured (*n* = 150 trap days and nights) in the three experimental plots.

Species	WQ		MQ		EQ	
	Trapped individuals	Proportion (%)	Trapped individuals	Proportion (%)	Trapped individuals	Proportion (%)
*Apodemus peninsulae*	13	56.5	29	65.9	21	56.8
*Apodemus draco*	5	21.7	9	20.5	7	18.9
*Sciurotamias davidianus*	3	13.0	6	13.6	9	24.3
*Apodemus agrarius*	2	8.7				
Total	23	100	44	100	37	100

WQ: study area located in the western Qinling Mountains; MQ: study area located in the middle Qinling Mountains; EQ: study area located in the eastern Qinling Mountains.

The seed crops of *Q. aliena* var. *acuteserrata* and *P. armandii*, respectively, in each plot were: 30.58±4.95 m^−2^ and 14.28±2.94 m^−2^ in MQ; 26.60±5.16 m^−2^ and 6.30±1.29 m^−2^ in EQ; 7.73±1.71 m^−2^ and 5.68±1.03 m^−2^ in WQ (mean ± SE). There were significant differences in seed crops among three plots for each of these two species (*P*<0.001, respectively).

### Removal rates of the two seed species at seed stations

**Figure 2:**
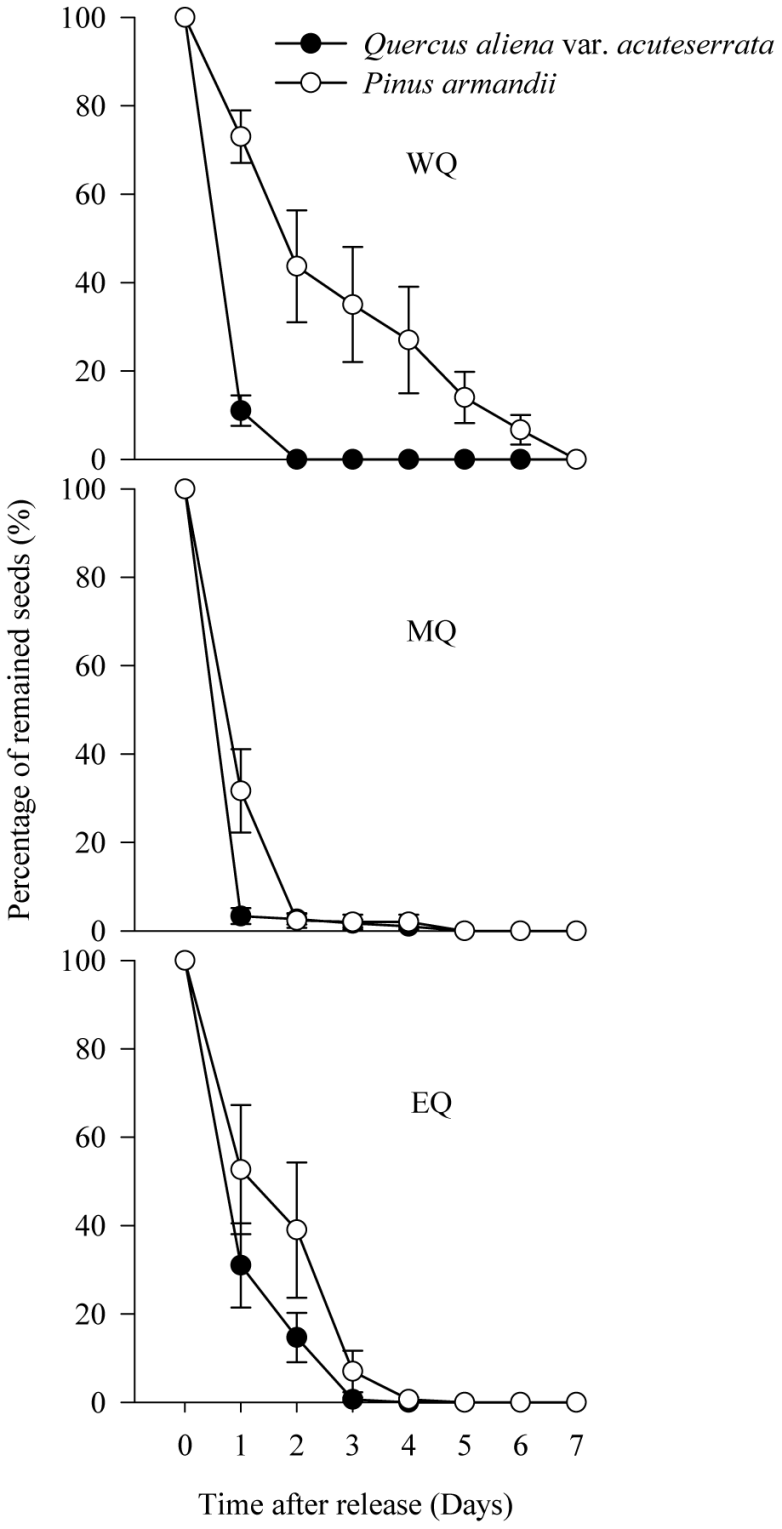
Seed removal rates of *P. armandii* and *Q. aliena* var. *acuteserrata* after deposition in the seed stations in the three experimental plots. WQ: study area located in the western Qinling Mountains; MQ: study area located in the middle Qinling Mountains; EQ: study area located in the eastern Qinling Mountains. Data are expressed as mean ± SE.

The investigations of *P. armandii* and *Q. aliena* var. *acuteserrata* seed dispersal demonstrated that 100% of the tagged seeds were removed by small rodents within seven days after their release in all plots (Fig. 2). Cox regression analysis detected no significant difference in the seed removal rates of the two species in all plots (*Wald* = 3.923, *df* = 1, *P* = 0.051 in WQ; *Wald* = 0.010, *df* = 1, *P* = 0.922 in MQ; *Wald* = 0.254, *df* = 1, *P* = 0.614 in EQ).

### Seed fates

**Figure 3:**
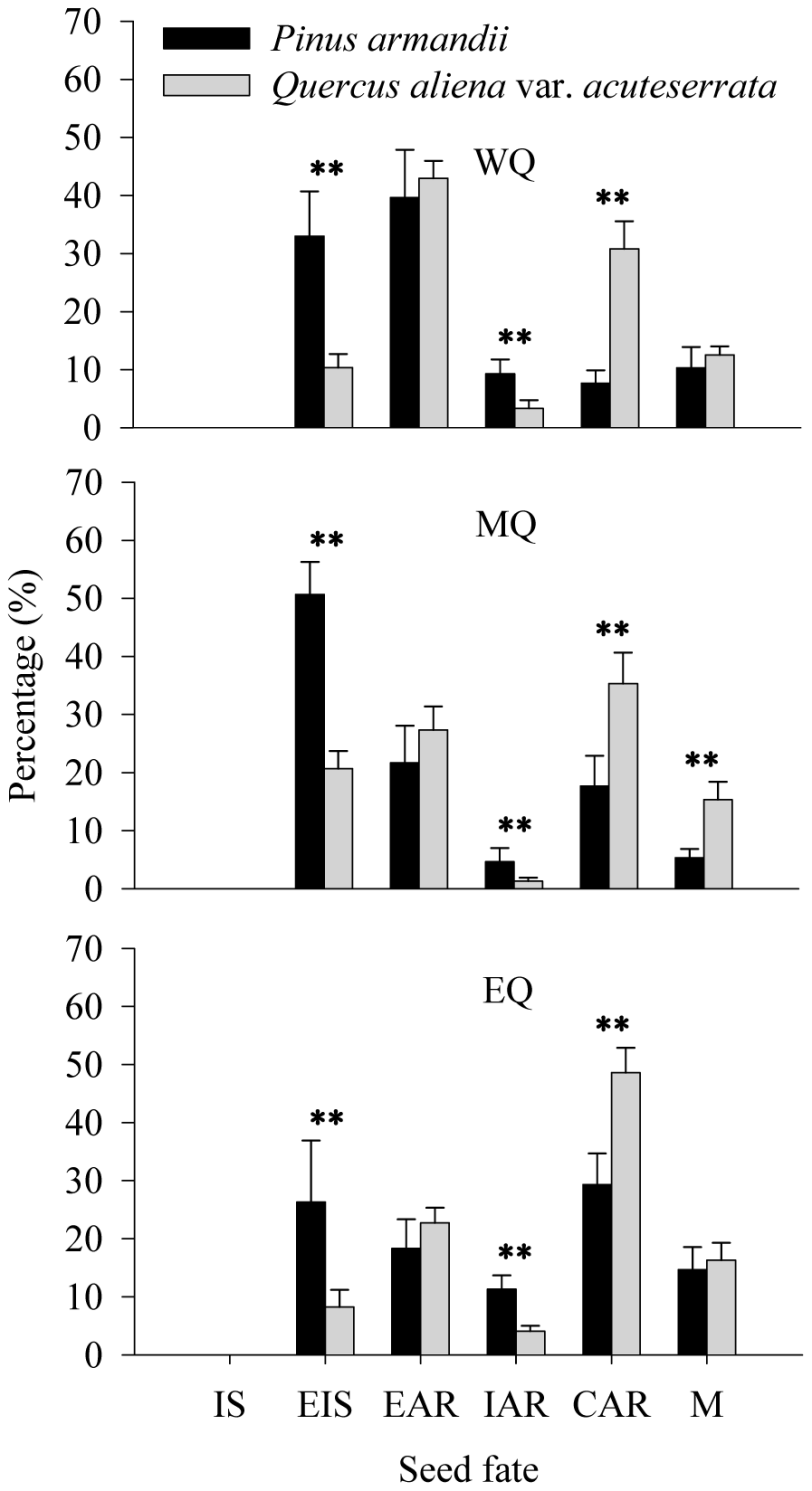
Fates of *P. armandii* seeds and *Q. aliena* var. *acuteserrata* acorns after dispersal by small rodents in the three experimental plots. WQ: study area located in the western Qinling Mountains; MQ: study area located in the middle Qinling Mountains; EQ: study area located in the eastern Qinling Mountains. IS: in situ; EIS: eaten in situ; IAR: intact after removal; EAR: eaten after removal; CAR: cached after removal; M: missing. Data are expressed as mean ± SE. **: statistically significant difference between the tree species (*P*<0.01).

Significantly more *P. armandii* seeds were eaten in situ (EIS) than *Q. aliena* var. *acuteserrata* seeds in all plots (33.0% compared to 10.4% in WQ, 50.7% compared to 20.7% in MQ, and 26.3% compared to 8.3% in EQ) (all *P*<0.001), while more *Q. aliena* var. *acuteserrata* seeds were cached after removal (CAR) in all plots (30.8% compared to 7.7% in WQ, 35.3% compared to 17.7% in MQ, and 48.6% compared to 29.3% in EQ) (all *P*<0.001) (Fig. 3). The eaten in situ (EIS), cached after removal (CAR), and eaten after removal (EAR) seed fate proportions were also affected significantly by plot (EIS: *F* = 3.676, *df* = 2, *P* = 0.032; CAR: *F* = 18.380, *df* = 2, *P*<0.001; EAR: *F* = 11.032, *df* = 2, *P*<0.001) (Fig. 3), while there were no significant interactions between seed species and plot (EIS: *F* = 0.526, *df* = 2, *P* = 0.594; CAR: *F* = 1.729, *df* = 2, *P* = 0.188; EAR: *F* = 0.579, *df* = 2, *P* = 0.564).

### Seed dispersal distance

**Figure 4:**
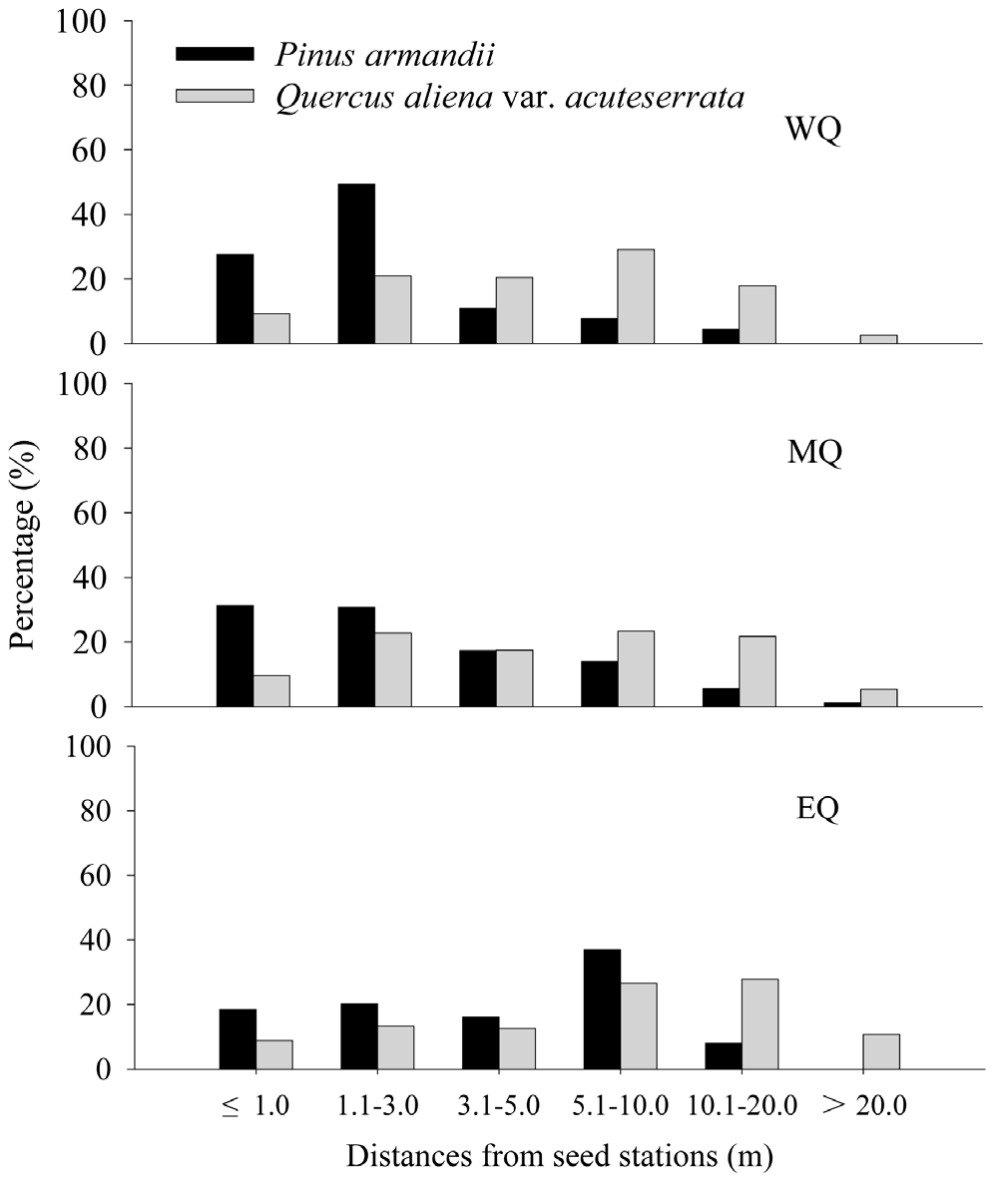
Distance distributions of *P. armandii* seeds and *Q. aliena* var. *acuteserrata* acorns in the three experimental plots. WQ: study area located in the western Qinling Mountains; MQ: study area located in the middle Qinling Mountains; EQ: study area located in the eastern Qinling Mountains.

Most of the seeds were dispersed less than 20 m in all plots (Fig. 4). The average dispersal distance was affected significantly by seed species (*F* = 158.215, *df* = 1, *P*<0.001) and plot (*F* = 14.082, *df* = 2, *P*<0.001) (Fig. 4), while the interaction between seed species and plot was not significant (*F* = 0.451, *df* = 2, *P* = 0.637). The average dispersal distances of *Q. aliena* var. *acuteserrata* seeds (6.98±0.40 m in WQ; 7.31±0.46 m in MQ; 9.41±0.58 m in EQ) were much greater than those of *P. armandii* (2.63±0.23 m in WQ; 3.19±0.33 m in MQ; 4.66±0.31 m in EQ) in all plots (all *P*<0.001). The maximum dispersal distances for *Q. aliena* var. *acuteserrata* and *P. armandii* seeds were 30.0 m and 17.1 m in WQ, 31.9 m and 11.0 m in MQ, and 35.4 m and 13.1 m in EQ, respectively. Only a few *P. armandii* seeds were transported into the *Q. aliena* var. *acuteserrata* stands in all plots (2.6% in WQ, 2.0% in MQ, and 1.3% in EQ). By contrast, over 30% of the *Q. aliena* var. *acuteserrata* seeds were transported into the *P. armandii* stands in all plots (36.7% in WQ, 35.0% in MQ, and 30.3% in EQ).

### Survival of cached seeds

A total of 46, 53, and 73 seeds of *Q. aliena* var. *acuteserrata* in primary caches were found to be recovered and subsequently re-cached in WQ, MQ and EQ, respectively, which further extended the cache distributions and the mean dispersal distances increased from 6.98 m for primary caches to 11.10 m for secondary caches in WQ (n = 46), from 7.31 to 9.62 m in MQ (n = 53) and from 9.41 to 13.93 m in EQ (n = 73). By contrast, almost all of *P. armandii* seeds in primary caches were found to be recovered and subsequently consumed. Only seeds of *Q. aliena* var. *acuteserrata* survived during our final survey. The total numbers of scatter-hoarded *Q. aliena* var. *acuteserrata* seeds that remained under the canopy were 22 in WQ, 29 in MQ, and 37 in EQ for the *P. armandii* forest, while the total numbers of seeds that remained under the canopy in the *Q. aliena* var. *acuteserrata* forest were 21 in WQ, 15 in MQ, and 35 in EQ. During spring 2013, a few *Q. aliena* var. *acuteserrata* seedlings emerged from the tagged seeds within the study area, i.e., under the canopy of *P. armandii* forest: one seedling in WQ, one seedling in MQ, and three seedlings in EQ; under the canopy of *Q. aliena* var. *acuteserrata* forest: one seedling in WQ, no seedlings in MQ, and three seedlings in EQ.

## Discussion

In our study, the rapid seed removal of *P. armandii* and *Q. aliena* var. *acuteserrata* from the seed stations and the lack of difference between the two species demonstrate the importance of small rodents for the seed dispersal effectiveness in the Qinling Mountains. Our observations agree with previous studies where fallen seeds were removed rapidly by rodents [14], [18], [29], [34]–[37].

We found different patterns of seed predation and dispersal in *Q. aliena* var. *acuteserrata* and *P. armandii*, which were both affected by small rodents. More seeds of *P. armandii* were eaten by small rodents, whereas *Q. aliena* var. *acuteserrata* producing relatively heavy seeds were more frequently dispersed by animals. Small rodents tended to scatter-hoard more *Q. aliena* var. *acuteserrata* seeds in all study sites. Previous studies have indicated that basic seed traits (e.g., seed size, nutrition content, and defensive secondary compounds, etc.) may be primary factors that affect the eating and caching strategies of rodents [15], [36], [38]–[39]. Seed size is considered to be a decisive factor for scatter-hoarding rodents in the choice between seed predation and caching [39]. Previous quantitative studies have suggested that small seeds are more likely to be eaten immediately, whereas large acorns are more likely to be cached by rodents for future use [34]–[37], [40]–[41]. Clearly, our results support the idea that the role of rodents during tree seed removal vary with tree species, although they are mainly dependent on seed size [42].

We also found that the seed dispersal distance was affected significantly by seed species, i.e., larger seeds were dispersed longer distances than small seeds. Our results clearly support the hypothesis of Jansen et al. [40] that larger seeds are dispersed further from their parent trees (or seed stations). Over 30% of the released acorns were moved into the *P. armandii* stands, whereas *P. armandii* seeds were rarely dispersed into the *Q. aliena* var. *acuteserrata* stands. These findings agree with other studies, which have shown that oaks can colonize the understory of pine forests via jay- or rodent-mediated dispersal of acorns [10], [11]. Our data support the previous prediction that *Pinus* species are pioneer species that are usually replaced by late successional *Quercus* species [9], [19]. Therefore, our results strengthened the hypothesis that rodent-mediated seed dispersal of *Q. aliena* var. *acuteserrata* into *P. armandii* stands facilitates the succession of the oak-pine mixed forests in Qinling Mountains, China.

*Q. aliena* var. *acuteserrata* is known to establish successfully in the *P. armandii* forest in Qinling Mountains [21], [26]; however, only a few *Q. aliena* var. *acuteserrata* seedlings germinated from our tagged seeds in the study area. Seed crop may have an important impact on seed survival and subsequent establishment [41], [43]–[44]. Our study indicated that seed fate and seed survival were different between plots, reflecting the spatial effects on seed dispersal and seed predation [45]. Previous studies have shown that scatter-hoarding and dispersal distances are enhanced during mast years compared with those during non-mast years (e.g. *Pinus* species and *Prunus armeniaca*) (predator dispersal hypothesis) [43]–[44]. After scatter-hoarding, seed survival and subsequent establishment were also higher in mast years, for example *Pinus* species [43] and *Carapa procera* [41]. Moreover, rodent population fluctuation may affect seed predation and seed dispersal [46]. Xiao et al. [47] argued that the predator satiation hypothesis, rather than predator dispersal hypothesis, provides a better mechanism for predicting seed dispersal and seed survival in animal-dispersed plants, because increasing seed production reduces seed dispersal but improves pre-dispersal seed survival. Despite three experimental plots with different seed crops established in the Qinling Mountains, future studies are needed to better understand how community-level seed abundance interacts with seed predators to predict seed dispersal and seed survival in animal-dispersed plants [47].

Previous studies have shown that oak seedlings have a similar abundance to pine seedlings in the pine forests [48] whereas pine seedlings are rarely found in oak forests in the mosaic distributions region between oak and pine forests [21], [26]. During the competition with pine regeneration, oaks exhibit an age advantage over pines and this advantage can be enhanced by the rapid growth of oaks under canopy shelter [10]. In addition, oak are shade tolerant in the seedling stage and can be established earlier than pine in the relatively dense old pine stands [10]. Both small-seeded animal-dispersed and wind-dispersed species (e.g., *Pinus ponderosa*, *Pinus contorta*, and *Pinus jeffreyi*) often depend on gaps for establishment [49]–[51]. A number of these species have dormant seeds that wait for a new gap to form, but dormancy also involves the risk of high attrition (e.g., the loss of dormant seeds to animals, fungi, and deep burial) [51]. Thus, *Quercus* species seem to have a higher competitive ability compared with *Pinus* species, which is supported by their current distribution in northern China [52].

In summary, we found that *P. armandii* had limited seed dispersal and experienced heavy predation by small rodents, whereas *Q. aliena* var. *acuteserrata* colonized the understory of pine forests and established successfully via the rodent-mediated dispersal of acorns. Although natural forest regeneration and succession may be influenced by many factors (e.g., post-dispersal seed survival) [8], [31], [53], [54], our study highlights the importance of the dispersal behavior of small rodents and their potential contribution to the invasion of oak into pine forests, better explaining the succession patterns in oak-pine mixed forests in the Qinling Mountains of China.
